# Department of Agriculture Discusses Objectionable Labeling for Medicinal Preparations

**Published:** 1914-11

**Authors:** 


### Abstracts and Selections.

### Department of Agriculture Discusses Objectionable Labeling for Medicinal Preparations.

In answer to many inquiries as to proper labeling for medicinal preparations to comply with the Food and Drugs Act as amended, the Department of Agriculture, through the Bureau of Chemistry, has issued the following suggestions to makers and proprietors of medicinal preparations:

1. *Claims of Therapeutic Effects.*—A preparation cannot be properly designated as a specific, cure, remedy, or recommended as infallible, sure, certain, reliable or invaluable, or bear other promises of benefit unless the product can as a matter of fact be depended upon to produce the results claimed for it. Before making any such claim the responsible party should carefully consider whether the proposed representations are strictly in harmony with the facts; in other words, whether the medicine in the light of its composition is actually capable of fulfilling the promises made for it. For instance, if the broad representation that the product is a remedy for certain diseases is made, as, for example, by the use of the word "remedy" in the name of the preparation, the article should actually be a remedy for the affections named upon the label under all conditions, irrespective of kind and cause.

2. *Indirect Statements.*—Not only are direct statements and representations of a misleading character objectionable, but any suggestion, hint, or insinuation, direct or indirect, or design or device that may tend to convey a misleading impression should be avoided. This applies, for example, to such statements as "has been widely recommended for," followed by unwarranted therapeutic claims.

3. *Indefinite and Sweeping Terms.*—Representations that are unwarranted on account of indefiniteness of a general sweeping character should be avoided. For example, the statement that a preparation is "for kidney troubles" conveys the impression that the product is useful in the treatment of kidney affections generally. Such a representation is misleading and deceptive unless the medicine in question is actually useful in all of these affections. For this reason it is usually best to avoid terms covering a number of ailments, such as "skin diseases, kidney, liver, and bladder affections," etc. Rheumatism, dyspepsia, eczema, and the names of many other affections are more or less comprehensive, and their use under some circumstances would be objectionable. For example, a medicine should not be recommended for rheumatism unless it is capable of fulfilling the claims and representations made for it in all kinds of rheumatism. To represent that a medicine is useful for rheumatism, when as a matter of fact it is useful in only one form of rheumatism, would be misleading; such statements as "for some diseases of the kidney and liver," "for many forms of rheumatism," are objectionable, on account of indefiniteness.

Names like "heart remedy," "kidney pills," "blood purifier," "nerve tonic," "bone liniment," "lung balm," and other terms involving the names of parts of the body are objectionable for similar reasons.

4. *Testimonials.*—Testimonials, aside from the personal aspect given them by their letter form, hold out a general representation to the public for which the party doing the labeling is held to be responsible. The fact that a testimonial is genuine and honestly represents the opinion of the person writing it does not justify its use if it creates a misleading impression with regard to the results which the medicine will produce.

No statement relative to the therapeutic effects of medicinal products should be made in the form of a "testimonial" which would be regarded as unwarranted if made as a direct statement of the manufacturer.

5. *Refund Guaranteed.*—Statements on the labels of drugs guaranteeing them to cure certain diseases or money refunded may be so worded as to be false and fraudulent and to constitute misbranding. Misrepresentations of this kind are not justified by the fact that the purchase price of the article is actually refunded as promised.

---

A new medicine glass cover is a saucer with a fluted rim, marked with the hours and quarters, to hold a spoon in such a position as to indicate the time for the next dose.

